# Secretomes from metastatic breast cancer cells, enriched for a prognostically unfavorable LCN2 axis, induce anti-inflammatory MSC actions and a tumor-supportive premetastatic lung

**DOI:** 10.18632/oncotarget.26903

**Published:** 2019-04-30

**Authors:** Kayla J. Meade, Francesca Sanchez, Analine Aguayo, Nathalie Nadales, Sarkis G. Hamalian, Toni L. Uhlendorf, Lisa R. Banner, Jonathan A. Kelber

**Affiliations:** ^1^Department of Biology, California State University Northridge, Northridge CA 91330, USA; ^*^These authors contributed equally to this work

**Keywords:** premetastatic niche, tumor microenvironment, metastasis, breast cancer secretomes, LCN2

## Abstract

Cancer metastasis is responsible for the clear majority of cancer-related deaths. Survival and expansion of cancer cells at secondary sites requires that these premetastatic microenvironments be primed by primary tumor cells and their secreted factors. Efforts to date have been limited by immune-deficient *in vivo* models and/or the need for finely-tuned analysis time points that reduce contributions from early-disseminating cancer cells. In this regard, we developed a tumor cell-free syngeneic breast cancer model for characterizing tumor cell secretome-mediated reprogramming of premetastatic tissues. We demonstrate that secretomes from metastatic breast cancer cells differentially regulate the lung and brain, promoting a tumor-supportive lung microenvironment with both elevated CD73 expression and decreased TNFα expression. Using *in vitro* models of CD73-positive mesenchymal stem cells (MSCs) and macrophages/monocytes, we tested whether MSCs can mediate anti-inflammatory effects of metastatic breast cancer cells. Notably, conditioned media from metastatic Py230 cells reprogrammed the secretomes of MSCs toward an anti-inflammatory state. Mining transcriptome data from Py8119 and Py230 cells revealed a lipocalin 2 (LCN2) axis that is selectively expressed in the metastatic Py230 cells, predicts poor breast cancer patient survival and is elevated in circulating serum of mice chronically treated with conditioned media from Py230 cells. Taken together, these results establish the utility of an immune-competent tumor cell-free model for characterizing the mechanisms of breast cancer cell priming of the premetastatic niche, demonstrate that MSCs can mediate the anti-inflammatory effects of metastatic breast cancer cells and substantiate LCN2 as a promising therapeutic target for blocking breast cancer progression.

## INTRODUCTION

Metastasis is the major cause of most cancer-related deaths [1]. This is attributed to the metastatic spread of cells from the primary tumor to other sites such as the bone [2], lung [3], liver [4] and brain [5]. These metastatic sites must first be primed and acquire tumor-permissive properties to support the growth of the metastatic cancer cells [6]. Prior to tumor cell arrival, these potential sites of metastasis are termed the “premetastatic niche” and include a diverse profile of cells and molecules susceptible to pro-tumorigenic reprogramming. The concept of the premetastatic niche comes from the idea of the “seed and soil” hypothesis that defines the seed as cells from the primary tumor that colonize the “soil”, or specific organs, that have been prepared to support their growth [7].

During priming of the premetastatic niche, the primary tumor communicates with this distant microenvironment through secretion of biomolecules and extracellular vesicles into circulation. This results in the recruitment and modification of its cellular and molecular composition, not limited to macrophages and mesenchymal stem cells (MSCs), which have been reported to migrate to the newly tumor-primed environments [6, 8].

Both macrophages and bone marrow derived MSCs are recruited to the premetastatic niche and can aid in further recruitment of other cells to this new environment [9]. Also, MSCs are shown to recruit macrophages to the tumor site [10] and stimulate macrophage polarization further driving tumor progression [11]. Macrophages can adopt one of several fates that can either promote tumorigenesis (M2 polarization states produce anti-inflammatory factors) or inhibit it (M1 polarization states produce pro-inflammatory factors) [12].

Although some of the processes by which the primary tumor primes the premetastatic niche have been described, work to date has been predominantly limited to immune-deficient mouse models and the mechanisms by which various resident or newly recruited cell populations may interact during the premetastatic niche reprogramming phase of tumor progression remain poorly understood. Here, we sought to establish a tumor cell-free, immune-competent mouse model for evaluating how secretomes from metastatic versus non-metastatic breast cancer cells differentially remodel the premetastatic niche toward a tumor-supportive state. We further evaluate the expression of inflammation and MSC markers in brain and lung tissue. Finally, we consider how MSCs educated by metastatic breast cancer cells may be differentially reprogramed toward pro- or anti-inflammatory states. Our *in vivo* and *in vitro* data suggest that metastatic breast cancer cell secretomes may induce MSC-macrophage crosstalk during premetastatic niche reprogramming toward a tumor-supportive state. Our data also provide evidence for a role of lipocalin 2 (LCN2) during this premetastatic niche priming.

## RESULTS

### Metastatic PyMT breast cancer cell secretomes reduce pro-inflammatory TNFα and maintain CD73 expression levels in mouse lung

**Figure 1:**
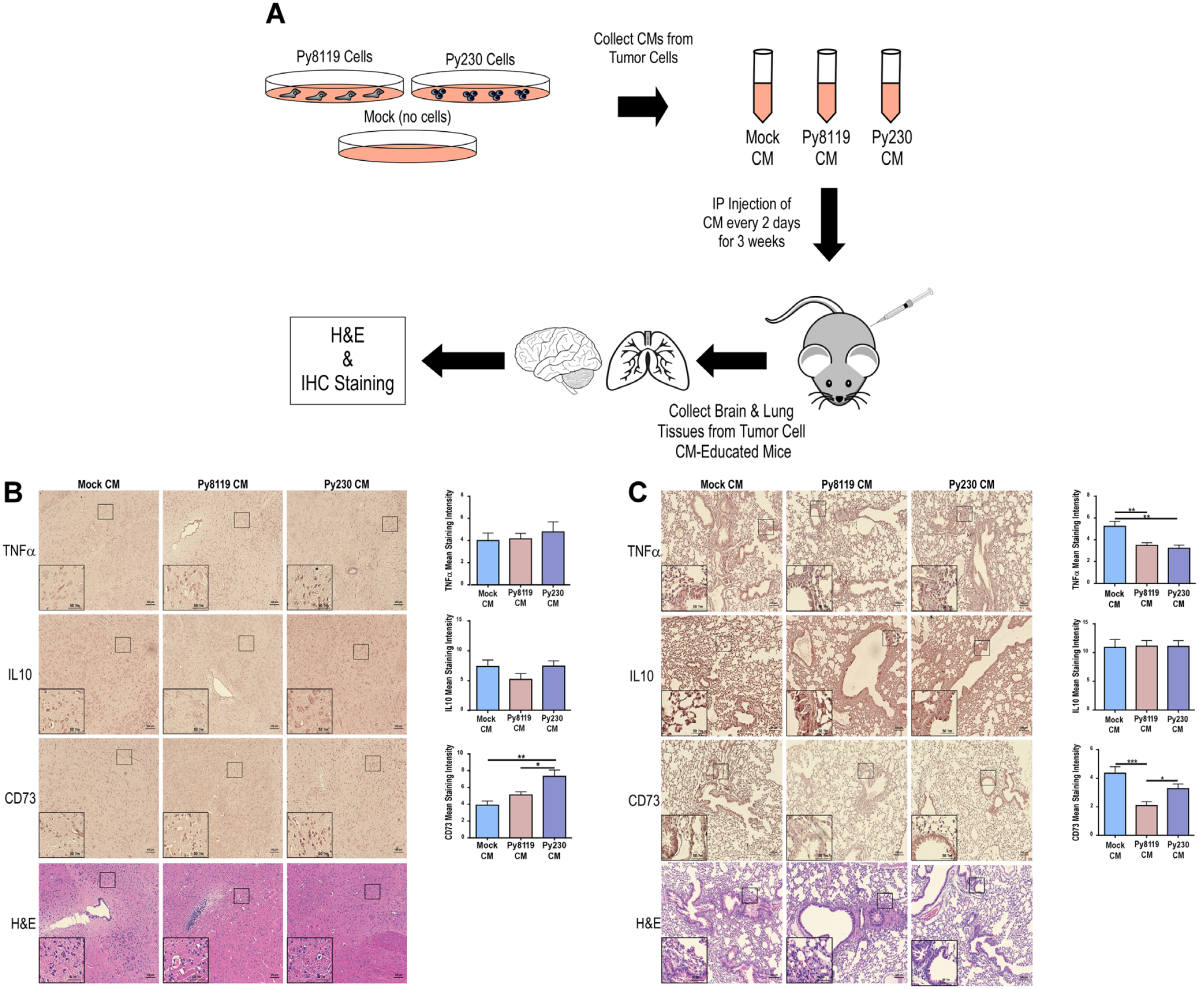
Metastatic PyMT breast cancer cell secretomes reduce pro-inflammatory TNFα and maintain CD73 expression levels in mouse lung. (**A**) Experimental scheme to test the effects of metastatic (Py230) and non-metastatic (Py8119) PyMT breast cancer cell conditioned media on brain and lung tissues. (**B–C**) IHC for TNFα, IL10, and CD73 markers and H&E of mouse brain in B and lung in C under the various treatment conditions (Mock CM, Py8119 CM, and Py230 CM). IHC was quantified using ImageJ for mean staining intensity. ^*^, ^**^, and ^***^ represent *p*-values of <0.05, 0.01, and 0.001, respectively, as determined by a One-Way ANOVA test with multiple comparisons post-testing. *N* = 10 mice per treatment group. 100 μm and 50 μm scale bars represent full images and image inlays, respectively.

To date, studies of how primary tumor cells communicate with the premetastatic niche have been primarily restricted to human tumor cell xenografts in immune-compromised animal models or carefully-tuned time-course studies to evaluate remodeling of distant tissues prior to observable metastasis [13–15]. Thus, a need exists to establish an immune-competent tumor cell-free model to evaluate the differential premetastatic niche reprogramming effects of metastatic and non-metastatic breast cancer cell derivatives in order to identify new therapeutic strategies for improving the outcomes for breast cancer patients. Using the non-metastatic Py8119 and metastatic Py230 [16] PyMT breast cancer models, we set out to evaluate the effects of the secretomes of these breast cancer cells on remodeling the histology and reprogramming markers of inflammation and mesenchymal cell populations in lung and brain tissues. As shown in Figure 1A, serum-free, conditioned media (CM) was collected from *in vitro* cultures of these cell lines along with media incubated under the same conditions in the absence of cells (Mock CM). These CM samples were injected intraperitoneally (IP) into recipient C57BL/6J mice every other day for three weeks. Mice across all treatment groups were sacrificed and brain and lung tissue was collected, fixed and sectioned for hematoxylin and eosin (H&E) and immunohistochemistry (IHC) staining for IL10 (anti-inflammatory, tumor-promoting), TNFα (pro-inflammatory, anti-tumorigenic) and CD73 (mesenchymal stem cell marker, tumor-promoting). For comparison, effects of Mock CM versus PBS sham injections were also compared (Supplementary Figure 1A–1C). Notably, no gross or histological differences were observed between tissue samples in any of the treatment groups (Figure 1B and 1C, Supplementary Figure 1B–1C). However, brain CD73 expression levels were markedly increased in the Py230-educated brain tissues (Figure 1B). In contrast, both non-metastatic Py8119 and metastatic Py230 secretomes reduced anti-inflammatory TNFα expression while the Py8119 secretomes selectively decreased CD73 levels in lung tissue (Figure 1C). Additional staining for the proliferation marker Ki67 was done across tissues from Mock CM, Py8119 CM and Py230 CM treated mice. Interestingly, no significant differences were observed (Supplementary Figure 1D) suggesting that the increased staining for CD73 in the mouse brain (Figure 1B) or maintenance of CD73 staining in the mouse lung (Figure 1C) may be due to CD73-positive cell recruitment, differentiation of progenitor cells into CD73-positive cells or increased CD73 expression in the resident stromal cells, as opposed to expansion of CD73-positive cells.

### The secretomes of metastatic breast cancer cells promote a tumor-supportive environment in the mouse lung

**Figure 2:**
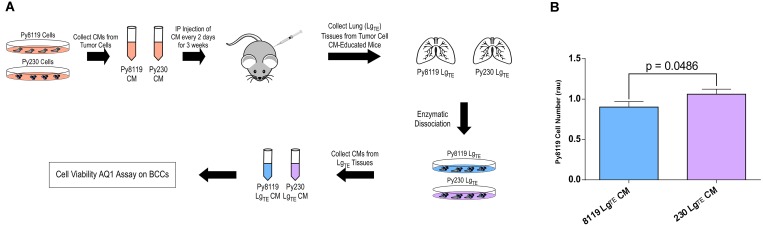
The secretomes of metastatic breast cancer cells promote a tumor-supportive environment in the mouse lung. (**A**) Experimental scheme to test the *ex vivo* effects of conditioned media from mouse lung tissues previously educated with either metastatic (Py230) and non-metastatic (Py8119) PyMT breast cancer cell conditioned media (Lg_TE_) on breast cancer cell (BCC) viability using the AqueousOne (AQ1) assay. (**B**) AqueousOne viability assay of Py8119 cells treated with conditioned media from Py8119- or Py230-educated lung tissue. *P*-value generated from student’s *T*-test. *N* = 13 mice per treatment group.

To maintain a tumor cell-free *in vivo* system and evaluate whether the lung tissue from mice educated with Py8119 versus Py230 were selectively reprogrammed to support tumor cell proliferation/survival, we further modified the experiment outlined in Figure 1A. First, freshly-collected lung tissue was gently enzymatically-digested. This dissociated tissue was then used to condition fresh serum-free media for subsequent *in vitro* analyses of breast cancer cell viability (Figure 2A). Notably, media conditioned with mouse lung tissue that had been chronically educated with metastatic Py230 secretomes selectively promoted the proliferation/survival of non-metastatic Py8119 breast cancer cells (Figure 2B), demonstrating that the molecular remodeling of this tissue, reported in Figure 1C, is associated with a tumor-supportive reprogramming of the lung microenvironment.

### Metastatic breast cancer cells reprogram MSCs toward an anti-inflammatory state

**Figure 3:**
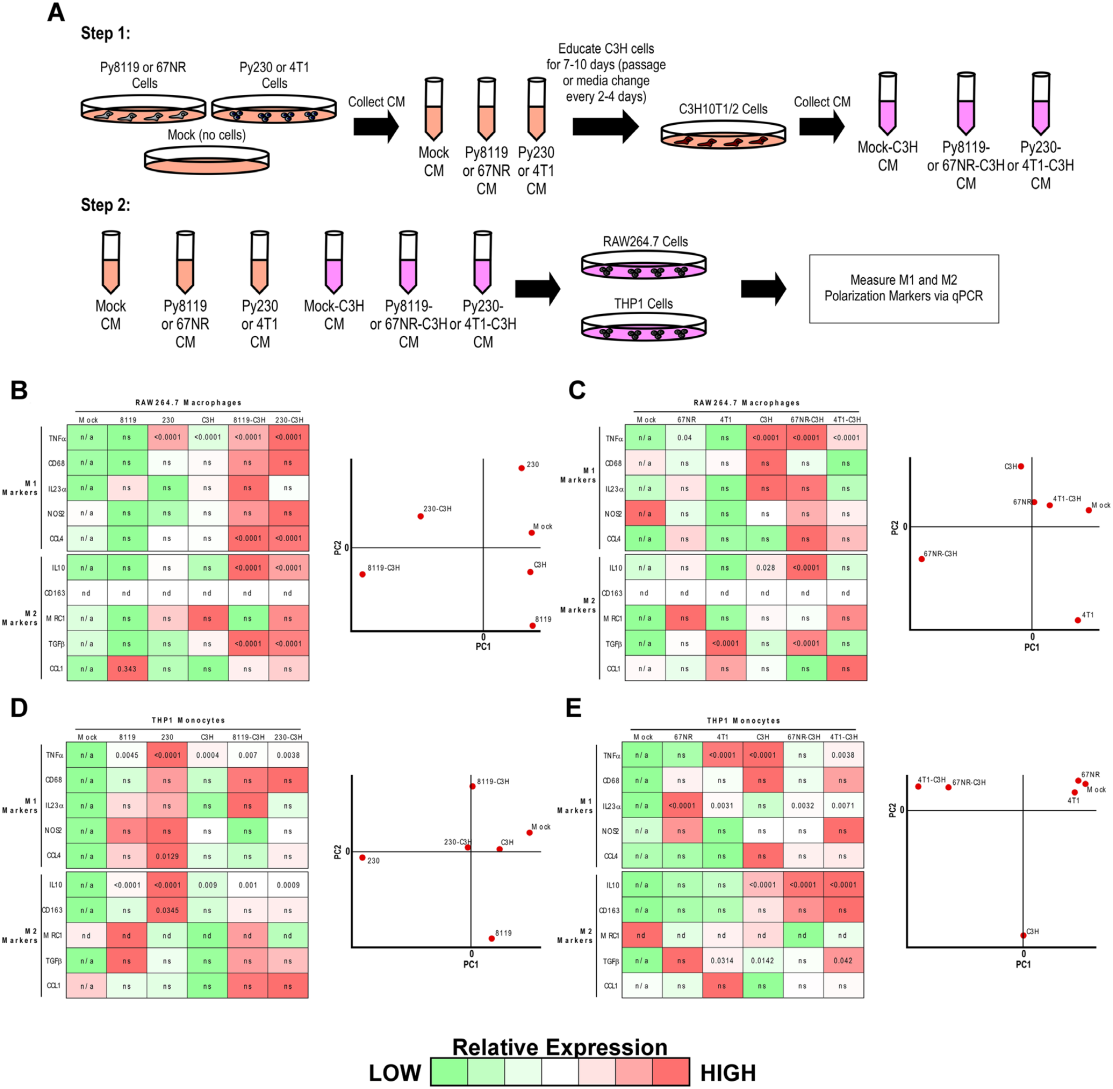
Metastatic breast cancer cells reprogram MSCs toward an anti-inflammatory state. (**A**) Experimental scheme to test the effect of conditioned medias from breast cancer cells or breast cancer cell conditioned media-educated MSCs on macrophage or monocyte gene expression profiles. (**B–E**) Gene expression heat maps and principal component analysis (PCA) plots from RAW246.7 cells in B and C, and THP1 cells in D and E for the six respective media treatment conditions outlined in A. Pseudo-colored heat maps were generated based upon qPCR relative quantification (RQ) values for each gene across all six samples normalized to the Mock conditioned media condition. One-way ANOVA was performed on each condition compared to Mock conditioned media and *p*-values < 0.05 were overlaid on the pseudo-colored heat map (ns = not significant, nd = not determined, n/a = not applicable). PCA plots were generated using Multiple Experiment Viewer (MeV) for all RQ data irrespective of statistical significance.

Given that the metastatic Py230 secretome remodeled the lung microenvironment toward a tumor-supportive, anti-inflammatory state while supporting the presence of MSCs, we next asked whether MSCs might directly mediate the anti-inflammatory effects of metastatic tumor cell secretomes. To do this, we first collected mock, non-metastatic and metastatic tumor cell secretomes from either the Py230/Py8119 or 4T1/67NR mouse breast cancer cell models. A fraction of these conditioned media samples was saved, while the other fraction was used to chronically treat mouse C3H10T1/2 MSCs. Additional conditioned media samples from these tumor-educated MSCs were also collected (Figure 3A). These conditioned media samples were then used to treat either the RAW264.7 macrophage or THP1 monocyte cell lines and purified RNA was analyzed by qPCR for a panel of five pro-inflammatory and five anti-inflammatory genes (Figure 3A). While the Py230, C3H, Py8119-C3H and Py230-C3H conditioned medias could significantly increase pro-inflammatory markers, Py230 cells were only able to significantly increase anti-inflammatory markers via a C3H MSC intermediate (Figure 3B, left and Supplementary Figure 2A). Principal Component Analysis (PCA) revealed that both Py230-C3H and Py8119-C3H treated macrophages are distinctly different from macrophages treated with the other four conditioned media samples (Figure 3B, right). In contrast, the metastatic 4T1 cells did not exhibit the same requirement for C3H MSCs as cell intermediates for inducing anti-inflammatory markers in macrophages (Figure 3C, left and Supplementary Figure 2B). As such, the 4T1-C3H treated macrophages shared more PCA similarity with the other samples (Figure 3C, right). Interestingly, the non-metastatic 67NR cells produce a highly pro-inflammatory state when C3H MSCs functioned as a cell intermediate (Figure 3C). In the THP1 monocyte system, the Py230 conditioned media alone produced a striking pro-inflammatory expression signature. Notably, when C3H MSCs acted as cellular intermediates, this signature became proportionate between the pro- and anti-inflammatory signatures and more similar to the effects of the Py8119-C3H conditioned medias (Figure 3D, left and Supplementary Figure 2C). In agreement with this observation, the Py230-treated monocytes stand out as a distinct population via PCA (Figure 3D, right). Finally, both 4T1 and 67NR conditioned medias were able to significantly increase anti-inflammatory markers via a C3H MSC intermediate (Figure 3E and Supplementary Figure 2D). Taken together, these results reveal both breast cancer cell model and macrophage differentiation state differences in tumor-induced inflammatory responses and support the possibility that the metastatic Py230 secretome may decrease inflammation within the lung microenvironment via MSC intermediates.

### Metastatic breast cancer cell secretomes increase MSC and macrophage/monocyte viability and migration

We further sought to evaluate the effects of mouse and human breast cancer cell secretomes alone on MSC, macrophage and monocyte viability and motility (Supplementary Figures 3A, 4A and 5A). Interestingly, only the metastatic mouse 4T1 breast cancer cell conditioned media had a pro-survival/proliferation effect on the C3H MSCs (Supplementary Figure 3B and 3D). In contrast, both non-metastatic and metastatic mouse and human breast cancer secretomes increased viability of macrophages and monocytes relative to Mock CM (Supplementary Figures 4B, 4D and 5B). Similarly, while only metastatic mouse Py230 and high-grade human CA1a breast cancer cell conditioned medias increased the motility of C3H MSCs in relation to their non-metastatic or low-grade cancer cell counter parts (Supplementary Figure 3C), macrophage motility was positively affected by both non-metastatic and metastatic mouse and human breast cancer cell conditioned medias (Supplementary Figure 4C). Finally, only the high-grade CA1a human breast cancer cell conditioned media positively affected anti-inflammatory IL10 markers in both macrophages (Supplementary Figure 4E), while both CA1h and CA1a conditioned medias upregulated IL10 and downregulated TNFα in monocytes (Supplementary Figure 5C). Interestingly, only the non-metastatic triple-negative MDA-MB-468 breast cancer cell conditioned media had a negative effect on both IL10 and TNFα expression in monocytes (Supplementary Figure 5C).

### LCN2 is up-regulated in metastatic PyMT breast cancer cells and associates with poor patient prognosis

**Figure 4:**
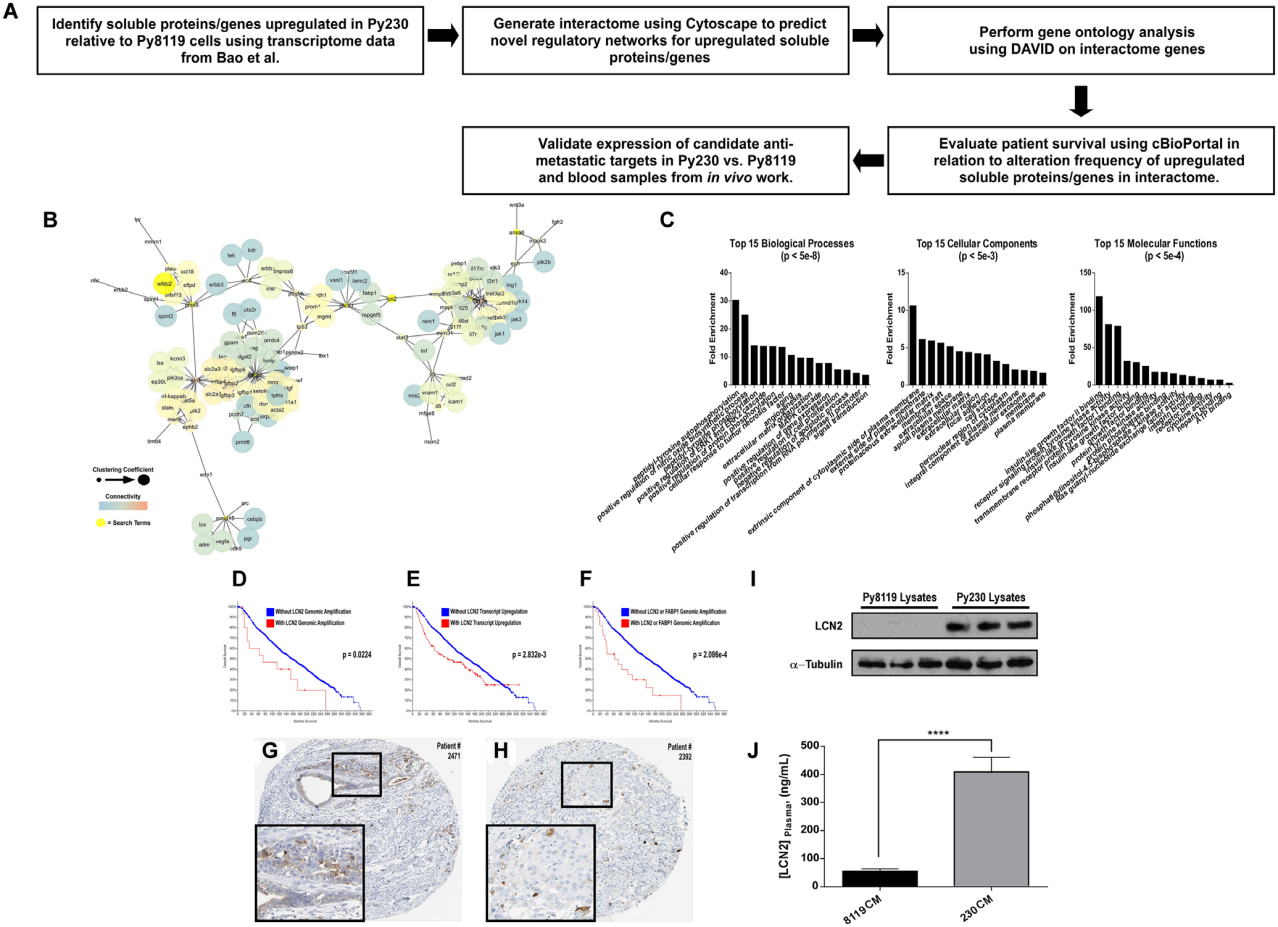
LCN2 is up-regulated in metastatic PyMT breast cancer cells and associates with poor patient prognosis. (**A**) Experimental scheme to determine soluble genes up-regulated in Py230 vs. Py8119 cells that have prognostic relevance. (**B**) Cytoscape interactome from soluble proteins/genes previously reported by Bao et al. to be upregulated in Py230 cells relative to Py8119 cells. (**C**) DAVID-generated gene ontology enrichment analysis for all nodes in the interactome in B. (**D–F**) Cancer BioPortal generated Kaplan-Meier survival curves for breast cancer patients in relation to when LCN2 is genomically amplified in D, is transcriptionally upregulated in E, or is co-amplified with FABP1 in F. (**G** and **H**) Human Protein Atlas IHC data for LCN2 and FABP1 in breast cancer tissue. (**I**) Immunoblot for LCN2 relative to loading control across triplicate protein lysates collected from either Py8119 and Py230 cells. (**J**) ELISA for LCN2 levels in circulating blood plasma from mice in the experiment outlined in Figure 2A. ^****^ represents a *p*-value of <0.0001 as determined by student’s *T*-test.

To identify possible mechanisms by which the metastatic Py230 secretomes may prime the premetastatic lung or brain, we identified 18 soluble factors from previous work [16, 17] that were up-regulated in Py230 cells relative to the non-metastatic Py8119 cells and devised a workflow to highlight any genes that when altered genomically showed prognostic significance in breast cancer patients (Figure 4A). First, we built a 133-node literature-based interactome using the Cytoscape Agilent Literature Search plugin and found that 9 of the initial 18 search terms represented nodes within the larger interactome (Figure 4B). As shown in Figure 4C, this interactome was enriched for tyrosine autophosphorylation, MAPK signaling and extracellular matrix organization Biological Process GOs; extracellular and cell surface Cellular Component GOs; and RTK, growth factor receptor and integrin signaling Molecular Function GOs. By screening these 9 genes for prognostic significance using patient-derived genomic and transcriptomic data available in the Cancer BioPortal, we discovered that LCN2 was the only gene in this list that when amplified (Figure 4D) or upregulated (Figure 4E) predicted poor patient survival. Additionally, when genomically co-altered, LCN2 and FABP1 (an LCN2 interacting node in Figure 4B) further decrease patient survival (Figure 4F) [17]. Importantly, both LCN2 and FABP1 are detectable in primary breast cancer patient samples (Figure 4G and 4H, respectively) [18–20]. We next validated expression of LCN2 in Py230 cell lysates relative to Py8119 cell lysates via immunoblot and confirmed selective expression in Py230 cells (Figure 4I). Finally, we tested blood serum samples from mice in experiments described in Figures 1A and 2A for circulating levels of LCN2 using an ELISA. Notably, chronic IP injections of Py230 conditioned media was sufficient to significantly elevate serum LCN2 levels by nearly 10-fold over that of the serum from the Py8119 conditioned media treated animals (Figure 4J).

### Model of potential mechanisms by which soluble factors from the primary breast tumors may induce an anti-inflammatory state within premetastatic tissues

**Figure 5:**
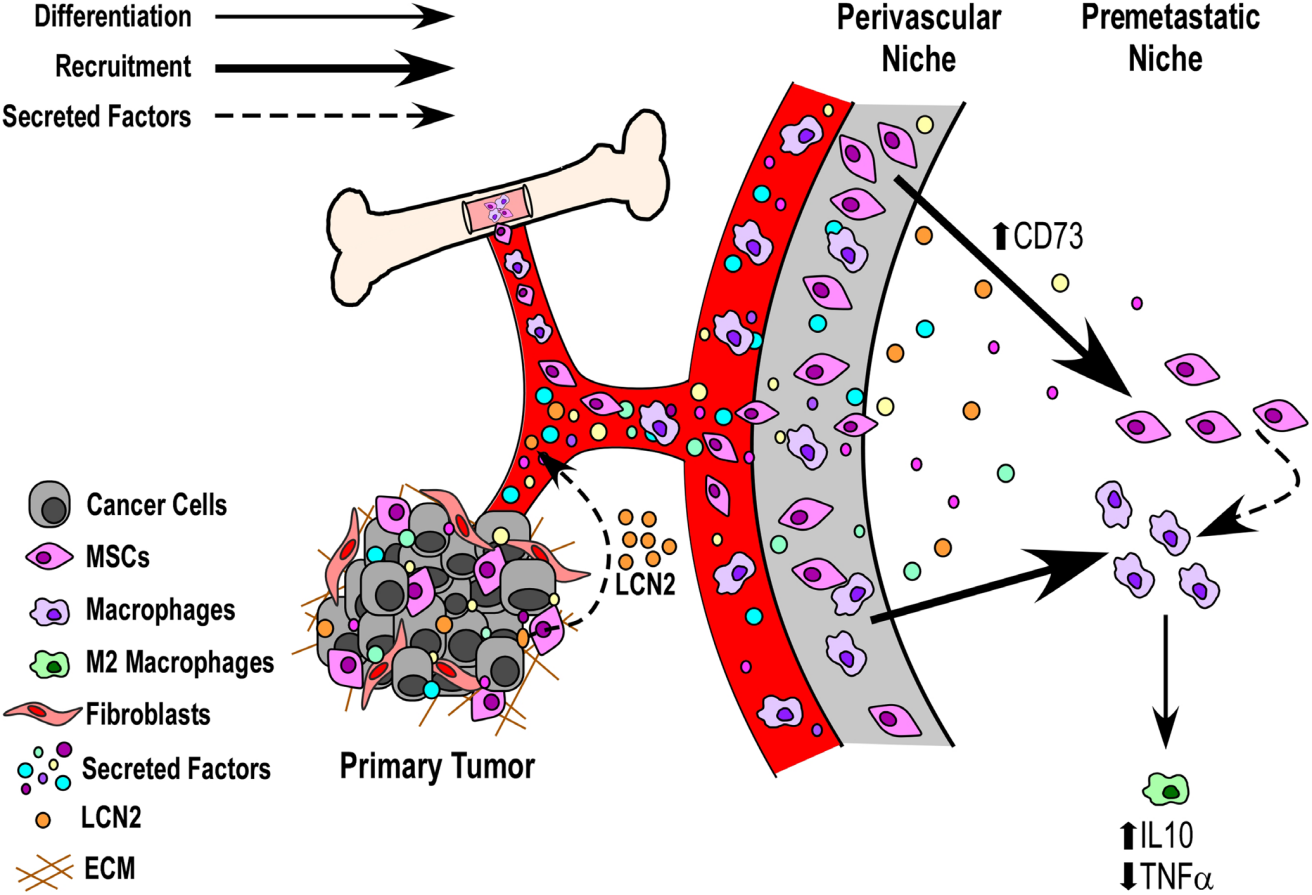
Model of potential mechanisms by which soluble factors from the primary breast tumors may induce an anti-inflammatory state within premetastatic tissues. Data presented herein suggest a potential role for primary tumor released soluble factors (e.g., LCN2) that can act via circulation and communicate with bone marrow derived cells (BMDCs) to prime the premetastatic niche toward a tumor-supportive state. Of interest for future work will be the analysis of whether such secreted factors act to remodel the premetastatic niche directly to recruit BMDCs (e.g., liberating mesenchymal stem cells from the perivascular niche) or whether they directly influence the recruitment and/or expansion of BMDCs from circulation.

Here, we provide a tumor cell-free, immune-competent PyMT model of breast cancer to analyze the effects of tumor cell secreted factors on the remodeling/reprogramming of the premetastatic niche. As shown in the Figure 5 schematic, secretomes of metastatic breast cancer cells that express high levels of secreted LCN2 (a marker for poor patient prognosis) increase systemic circulating levels of LCN2 and remodel the lung toward a tumor-permissive state, concurrent with increased CD73 (a marker of MSCs) expression and decreased TNFα (a pro-inflammatory marker) expression within the lung microenvironment. These *in vivo* data are supported by *in vitro* experiments demonstrating that metastatic breast cancer cells can preferentially induce anti-inflammatory states in macrophages via an MSC intermediate.

## DISCUSSION

The premetastatic niche is a complex and evolving environment that can be influenced by many systemic and tumor-specific factors. Growth factors, cytokines and cells can function to prime the premetastatic niche. For example, lysyl oxidase (LOX), a secreted collagen cross-linking enzyme, has been determined to have elevated expression in the premetastatic niche [6] and is able to increase tumor cell colonization during the metastatic cascade [21]. Exosomes from primary pancreatic tumor cells have also been reported to be taken up by liver cells and increase bone marrow derived macrophage recruitment and TGFβ expression leading to formation of a fertile premetastatic niche [22]. Although we know that many cell types, secreted factors, cell-to-cell interactions, and cell recruitment events are involved in priming the premetastatic niche, we do not fully understand how certain stromal cell types within this environment communicate with one another to potentiate this process. In this regard, we evaluated the expression of anti-inflammatory IL10, pro-inflammatory TNFα and MSC CD73 markers within the lung and brain niches in response to systemic conditioning with metastatic and non-metastatic breast cancer cell secretomes (Figure 1A), and characterized the influence of tumor-educated derivatives of these stromal cell types on one another *in vitro* (Figure 3A). In particular, the observation that only secretomes from metastatic cells both sustain CD73 and decrease TNFα expression in the lung (Figure 1C), while also preferentially functioning to increase the inflammatory state of macrophages in the absence of MSCs *in vitro* (Figure 3B and Supplementary Figure 2A), suggests that secreted factors from the primary tumor may function to recruit and reprogram MSCs toward an anti-inflammatory state within the premetastatic niche. It will be relevant for future studies to determine whether inhibition of anti-inflammatory markers or MSC recruitment into the lung can prevent complementary stromal cell recruitment and/or this altered inflammation profile, respectively, in response to metastatic Py230 cells, and whether this may reverse the tumor-supportive state within the Py230-educated lung (Figure 2).

The PyMT model of breast cancer has been previously used to study the microenvironmental influence on disease progression in an immune-competent microenvironment [23]. Previous studies using this model, determined that when the Axl receptor is lost in tumor cells they develop an anti-tumor immune response with enhanced sensitivity to immunotherapy [24]. Recent evidence also suggests local and systemic inflammation is a major inducer of metastatic spread. More specifically, other syngeneic tumor models have been used to determine that pro-inflammatory responses associated with primary tumor resection can lead to early relapse in breast cancer [25]. In order to prevent this relapse from occurring post-surgery, Retsky and colleagues determined that non-steroid anti-inflammatory therapies may be helpful [26]. It is interesting to consider these studies in light of our findings that only secretomes from metastatic Py230 breast cancer cells decrease pro-inflammatory TNFα while maintaining CD73 in the lung (Figure 1C). In contrast, TNFα and IL10 levels remained unchanged in the premetastatic brain of these same mice (Figure 1B). Importantly, these data support the notion that premetastatic niche priming mechanisms may occur via tissue-specific factors. It will be important for future studies to determine the effect that surgery or systemic inflammation may have on tissue-specific metastasis when otherwise non-metastatic breast cancer cells are allografted. While the importance of the systemic effect from a growing primary tumor (and its resection) represent essential aspects of cancer progression and metastasis, we provide evidence that relevant studies may also be carried out within the context of a tumor cell-free, immune-competent model of breast cancer as well (Figure 2). Combining this approach with *in vivo* metastasis studies that rely on tumor cell grafting (i.e., spontaneous metastasis) or intravenous injection (i.e., experimental metastasis) may help differentiate the influence of tumor cell intrinsic and extrinsic factors on the metastatic cascade, as well as address the potentially very early stages of metastasis, before a primary tumor may even be detectable.

Finally, it is interesting to note that while previous studies have identified LCN2 as a promising therapeutic target to abrogate progression and metastasis in breast cancer [27–32], other work has suggested that LCN2 is not necessary for metastasis [33]. In either case, previous studies have not evaluated the role of LCN2 on the remodeling or reprogramming of the premetastatic niche toward a tumor-permissive state. In agreement with a potential cooperative role for both FABP1 and LCN2, previous work has identified FABP1 as a marker for circulating tumor cells (CTCs) in patients with advanced stage cancers [34]. In this regard, it will be important for future studies to evaluate whether LCN2 is necessary and/or sufficient for promoting a tumor-supportive state within the premetastatic niche. It will also be important to evaluate whether FABP1-positive CTCs may preferentially localize to tissues that have been reprogrammed by LCN2. Ultimately, combining therapies that target this axis with other standard therapies may help improve tumor remission rates and patient survival.

## MATERIALS AND METHODS

### Cell culture

MDA-MB-468, RAW264.7 and C3H10T1/2 cells were cultured in DMEM-High glucose growth media supplemented with fetal bovine serum (FBS) and antibiotics. 4T1 and 67NR cells were cultured in RPMI-1640 growth media supplemented with FBS and antibiotics. BT549 cells were cultured in RPMI-1640 growth media supplemented with insulin, FBS and antibiotics. THP1 cells were cultured in RPMI-1640 growth media supplemented with 2-mercaptoethanol, FBS and antibiotics. MCF10CA1h and MCF10CA1a cells were cultured in DMEM F12 growth media supplemented with horse serum and antibiotics. Py230 cells were cultured in F-12K growth media supplemented with MITO+ Serum Extender, fetal clone serum (FCS) and antibiotics. Py8119 cells were cultured in F-12K growth media supplemented with FCS and antibiotics.

### Tumor cell conditioned medias

All mouse (67NR, 4T1, Py8119 and Py230) and human (MDA-MB-468, BT549, MCF10CA1h and MCF10CA1a) BCCs were plated in 10 cm cell culture dishes. After the breast cancer cells (BCCs) reached an ~85% confluency either DMEM-High glucose growth media supplemented with FBS and antibiotics or RPMI-1640 growth media supplemented with 2-mercaptoethanol, FBS and antibiotics was added. Equal amounts of both types of media were collected to account for treatment of RAW264.7 (needed DMEM), C3H10T1/2 (needed DMEM), and THP1 (needed RPMI-1640). Mock CM (media without cells) served as the control and was collected with each set of mouse and human breast cancer cell CM. After 48 hours, the CM was collected, centrifuged and stored at –80°C until needed. CM was centrifuged at 1.0 × g for 5 minutes in place of media filtration. Before the CM was used, it was brought to a pH of about 7.0 and diluted 1:1 with serum free DMEM-High glucose or RPMI-1640 growth media supplemented with antibiotics. For CM from tumor cell-educated mesenchymal stem cells (MSCs): C3H10T1/2 cells were plated in 10 cm cell culture dishes and conditioned with mouse breast cancer cell CM or Mock CM for 7–10 days. The CM was then removed and replaced with DMEM-High glucose growth media supplemented with FBS and antibiotics. After 48 hours, the CM from tumor cell-educated C3H10T1/2 was collected, centrifuged and stored at –80°C until needed. For *in vivo* injections, PyMT cells conditioned serum/antibiotic free RPMI-1640 growth media and processed in the same manner described above.

### Mouse model

Five-week-old C57BL/6J female mice were purchased from The Jackson Laboratory. Intraperitoneal injections (IP) began one week after the mice arrived. Mice were maintained and monitored upon receipt and through the entire experimental procedure in accordance with IACUC protocol #1516-018. Mice were injected every other day for a total of 3 weeks with 500 μL of Mock, Py8119 and Py230 CM (as shown in Figure 1A) or with only Py8119 and Py230 CM (as shown in Figure 2A). PBS was used as a sham injection. The CM was collected as previously described, with the exception of FBS and antibiotics addition. Before injection, each CM was handled as previously stated. After 3 weeks, mice were sacrificed, and their lungs and brains were removed, flash-frozen fresh, fixed/stored in 10% formalin/95% EtOH prior to histology/IHC analysis or stored in Hibernate A media prior to immediate enzymatic disassociation. Serum was also collected from mice IP injected with Py8119 and Py230 CM.

### Tissue conditioned medias

Lung tissue from tumor cell-CM educated (TE) mice were rinsed thoroughly with antibiotics and gently enzymatically disassociated with Collagenase/PBS solution for 1 hour. The tissue cell suspensions were plated in cell culture plates in serum-free RPMI-1640 growth media supplemented with antibiotics. After 72 hours, the tissue-conditioned media was then collected, centrifuged (1.0 × g for 5 minutes in place of media filtration) and stored at –80°C until needed. Protein concentration of Py8119 - and Py230-Lung (Lg_TE_) CM were determined with the use of the Bradford Assay. Proportional to total protein concentration in each conditioned media sample, the Lg_TE_ CM was appropriately diluted in RPMI-1640 growth media, supplemented with 2% FBS and antibiotics, then used in the CellTiter 96^®^ AQueous One Solution (Promega) cell viability/ assay with Py8119 cells.

### Extracellular matrix (ECM) protein coating

All ECM proteins were coated at a concentration of 5 μg/ml. Each cell line was plated on a different substrate based on the CM that was used and is outlined as follows. C3H10T1/2 cells were plated on collagen and laminin and RAW264.7 cells were plated on collagen and plastic when treated with 67NR and 4T1 CM. When conditioned with Py8119 and Py230 CM C3H10T1/2 cells were plated on collagen and laminin and RAW264.7 cells were plated on collagen and plastic for migration. Plastic was used for both cell lines for cell viability assays. C3H10T/12 and RAW264.7 cells were plated on fibronectin and plastic when conditioned with human CM. THP1 cells were plated on plastic for all conditions and assays.

### Cell viability assay

The CellTiter 96^®^ AQueous One Solution (Promega) was used in viability and proliferation experiments. RAW264.7, C3H10T1/2, and Py8119 cells were plated at 5e3 cells/mL in a 96-well plate. Cells were treated the next day with either mouse or human breast cancer cell CM (RAW264.7 and C3H10T1/2) or Lung-CM (Py8119). THP1 cells were plated into mouse and human breast cancer cell CM at 1e4 cells/mL in a 96-well plate. After 48, 72, and 96 hours post CM treatment, AQueous One Solution was added to each well. Absorbance was measured at 490 nm wavelength after 3 hours.

### Phase contrast microscopy

Images of cells were taken from 96-well plates after 96 hours post-treatment with breast cancer cell CM. Widefield-brightfield phase contrast images were taken using a Leica DMI6000 B inverted microscope at 20X magnification.

### Migration

RAW264.7 cells (2e4 cells/mL) and C3H10T1/2 cells (4e3 cells/mL) were plated in a 24-well plate. Cells were treated the next day with mouse and human breast cancer cell CM. The cells were imaged over the course of 24 hours, with images taken every 10 minutes. After 24 hours, the time-lapse images were quantified for displacement and velocity using FIJI and the TrackMate plug-in as previously described [35]. Displacement is the measure of linear movement by the cell during the entire time course (i.e., the length of a straight path from its starting coordinate to its ending coordinate). Velocity is calculated as the average velocity of the cell as it moves along its entire track length (in rare instances the track length can approach the displacement, but this is only when a cell moves in an almost perfectly straight line – traditionally, the displacement divided by track length is a measure of straightness or persistence of cell motility).

### mRNA purification, cDNA synthesis and quantitative polymerase chain reaction (qPCR)

RAW264.7 or THP1 cells were plated at 2e5 cells/mL in a 6-well plate. Cells were treated the next day with mouse and human breast cancer cell CM. After 48 hours, the cells were scraped and pelleted. The remainder of the qPCR protocol was carried out as previously described [35, 36]. qPCR-specific primers for all 10 genes of interest were purchased pre-made through IDT. The sequences are as follows:

#### Human

TNFα 5′ – TCAGCTTGAGGGTTTGCTAC; TNFα 3′ – TGCACTTTGGAGTGATCGG.

CD68 5′ – CCATGTAGCTCAGGTAGACAAC; CD68 3′ – CCACCTGCTTCTCTCATTCC.

IL23α 5′ – GATTTTGAAGCGGAGAAGGAGA; IL23α 3′ – GCTTCATGCCTCCCTACTG.

NOS2 5′ – GCAGCTCAGCCTGTACT; NOS2 3′ – CACCATCCTCTTTGCGACA.

CCL4 5′ – ACTGTCCTGTCTCTCCTCAT; CCL4 3′ – CTTCCTCGCGGTGTAAGAAA.

IL10 5′ – TCACTCATGGCTTTGTAGATGC; IL10 3′ – GCGCTGTCATCGATTTCTTC.

CD163 5′ – ATCCGCCTTTGAATCCATCTC; CD163 3′ – GTCCTCCTCATTGTCTTCCTC.

MRC1 5′ – CAAGTTGCCGTCTGAACTGA; MRC1 3′ – TATCTCTGTCATCCCTGTCTCT.

TGFβ 5′ – GTTCAGGTACCGCTTCTCG; TGFβ 3′ – CCGACTACTACGCCAAGGA.

CCL1 5′ – TCTGAACCCATCCAACTGTG; CCL1 3′ – GCAATCCTGTGTTACAGAAATACC.

#### Mouse

Tnfα 5′ – TCAGCTTGAGGGTTTGCTAC; Tnfα 3′ – TGCACTTTGGAGTGATCGG.

Cd68 5′ – CCATGAATGTCCACTGTGCT; Cd68 3′ – CACCTGTCTCTCTCATTTCCTT.

Il23α 5′ – TGAAGATGTCAGAGTCAAGCAG; Il23α 3′ – ACAAGGACTCAAGGACAACAG.

Nos2 5′ – CACTTCTGCTCCAAATCCAAC; Nos2 3′ – GACTGAGCTGTTAGAGACACTT.

Ccl44 5′ – GTCTCATAGTAATCCATCACAAAGC; Ccl4 3′ – CTCTCTCTCCTCTTGCTCGT.

Il10 5′ – TCACTCATGGCTTTGTAGATGC; Il10 3′ – GCGCTGTCATCGATTTCTTC.

Cd163 5′ – ATCCGCCTTTGAATCCATCTC; Cd163 3′ – GTCCTCCTCATTGTCTTCCTC.

Mrc1 5′ – CAAGTTGCCGTCTGAACTGA; Mrc1 3′ – TATCTCTGTCATCCCTGTCTCT.

Tgfβ 5′ – CCGAATGTCTGACGTATTGAAGA; Tgfβ 3′ – GCGGACTACTATGCTAAAGAGG.

Ccl1 5′ – GAAGCTCTTTCTTCAAGGTG; Ccl1 3′ – CCATGAAACCCACTGCCAT.

### Principal component analysis (PCA)

Normalized qPCR RQ values for all 10 gene transcripts across each of the 6 treatment group samples were input into WebMeV (Multiple Experiment Viewer, http://mev.tm4.org/#/welcome), a cloud-based application supporting analysis, visualization and stratification of genomic and transcriptomic data sets. PCA graphs were automatically generated to evaluate similarity between treatment group samples within each of the four experiments (Py-RAW, 4T1-RAW, Py-THP1 and 4T1-THP1).

### Immunohistochemistry (IHC)

Mouse lung and brain samples were sent to the UCLA Tissue Procurement Core Laboratory for paraffin embedding, tissue sectioning and H&E staining. The tissue was dehydrated and incubated in primary antibodies (IL10, TNFα and CD73) overnight. After overnight incubation, the tissues were washed and secondary antibodies from the Vectastain ABC-HRP Rabbit IgG and Vectastain Elite ABC-HRP Rat IgG kits were added to the corresponding tissue. The tissue was washed and the Vectastain reagent was added. Following the Vectastain reagent, the tissue was subjected to horseradish peroxidase until a noticeable color change was achieved. The tissue was then subjected to hematoxylin, then dehydrated and mounted using Permount. The tissue slides were imaged using a Zeiss microscope at 10X magnification.

### Bioinformatics analyses

Transcriptome data from Bao et al. [16] was accessed via the Geo2R (https://www.ncbi.nlm.nih.gov/geo/geo2r/) database portal (accession number [GEO: GSE61138]). Gene transcripts significantly elevated in the Py230 cells relative to the Py8119 cells were searched on GeneCards (https://www.genecards.org/) for their subcellular localization to identify the extracellular protein products of interest. The resulting 18 genes were input into the Cytoscape (https://cytoscape.org/) Agilent Literature Search plugin – max engine matches were set to 20, aliases were used, context was used, concept lexicon was not used and interaction lexicon was set to “limited.” All 133 unique interactome nodes were input into the DAVID Bioinformatics Resource v6.8 (https://david.ncifcrf.gov/summary.jsp) to identify Gene Ontologies (GOs) enriched among these interactome nodes. The 9 genes, for which transcripts were enriched in the Py230 cells, and that were also connected within this interactome were individually searched on Cancer BioPortal (https://www.cbioportal.org/) for associations between transcript upregulation or genomic alteration with poor patient survival in the largest of the available breast cancer datasets from Pereira et al. [17]. Since LCN2 was the only soluble factor represented in the interactome whose genomic amplification and transcriptional upregulation predicted poor patient survival, the four interacting notes of LCN2 in the Cytoscape interactome were also queried in Cancer BioPortal for independent and co-alteration (with LCN2) prognostic value. Subsequent analysis of patient tissue samples for LCN2 and FABP1 protein levels was completed using the Human Protein Atlas (https://www.proteinatlas.org/) to verify that LCN2 and FABP1 can be detected within primary breast cancer tissues.

### Western blotting

Cells were lysed with the RIPA Buffer, then rotated at 4°C for ~3 hours. Lysates were centrifuged, then protein concentration in the supernatant was determined with the use of the Bradford Assay. Lysate proteins were resolved on a 4–12% Bis-Tris gel 2 hours at 100 volts and then transferred to a nitrocellulose membrane. Immunoblotting was performed overnight at 4°C with anti-LCN2 (R&D Systems #AF1857) (1:800 dilution) and α-tubulin (ProSci #7597) (1:1000 diltion) in milk-based blocking solution. A dilution of 1:10,000 was used for all secondary antibodies (Promega).

### ELISA

Blood samples from mice were centrifuged at 2,000 rpm for 10 minutes in order to separate plasma from red blood cells. Plasma-LCN2 levels were then detected with the use of the Mouse Lipocalin-2/NGAL DuoSet ELISA (R&D Systems) according to manufacturer’s instructions.

## SUPPLEMENTARY MATERIALS


