# Supplementary material for: Secretomes from metastatic breast cancer cells, enriched for a prognostically unfavorable LCN2 axis, induce anti-inflammatory MSC actions and a tumor-supportive premetastatic lung
Source: Oncotarget. 2019 Apr 30;10(32):3027–39. doi: 10.18632/oncotarget.26903 (PMC6508963; doi:10.18632/oncotarget.26903)
Supplement: Supplementary file 1 [file oncotarget-10-3027-s001.pdf]

Secretomes from metastatic breast cancer cells, enriched for a prognostically unfavorable LCN2 axis, induce anti-inflammatory MSC actions and a tumor-supportive premetastatic lung

SUPPLEMENTARY MATERIALS

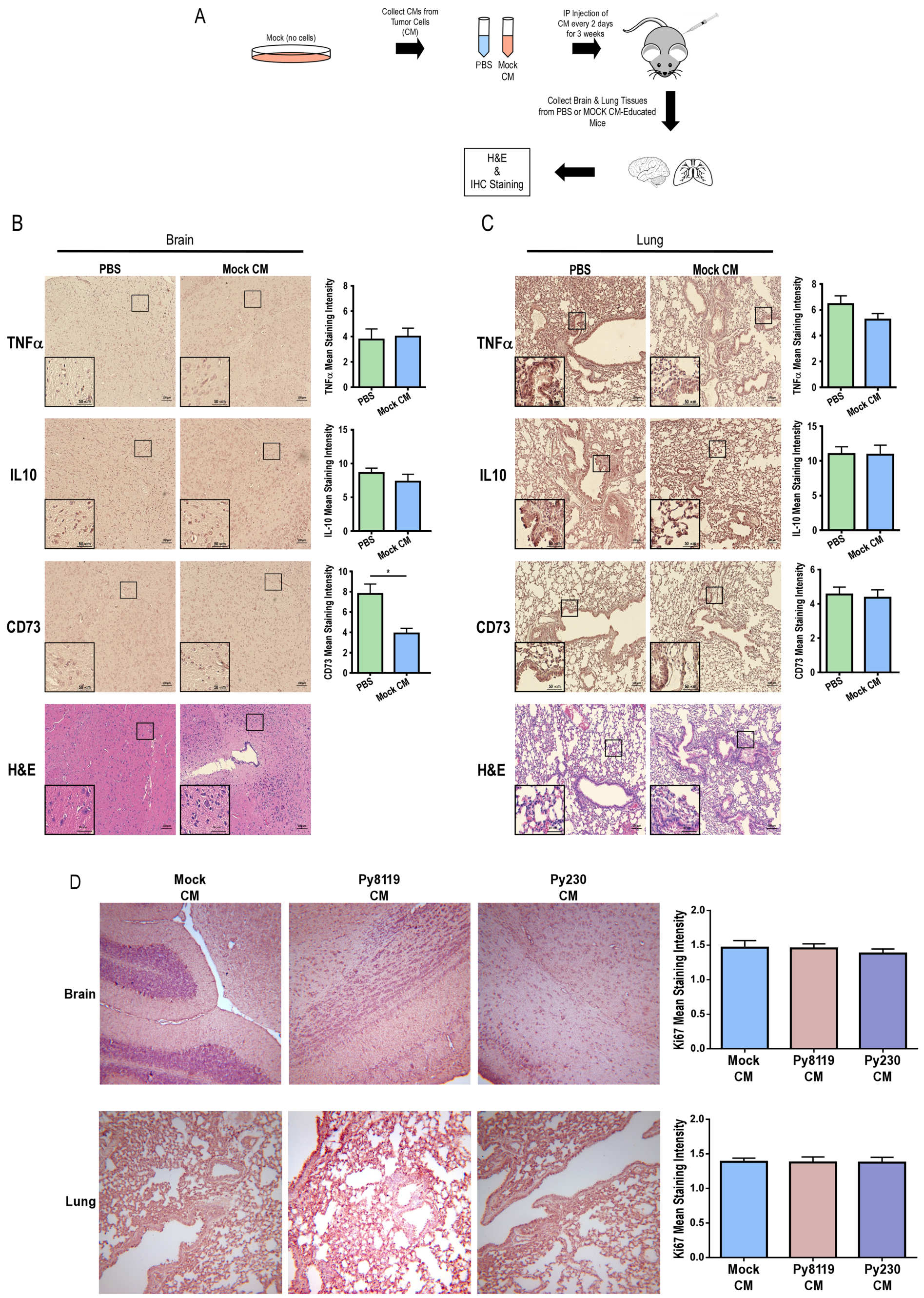

Supplementary Figure 1: Metastatic CM maintains IL10 and CD73 expression in the mouse brain. (**A**) Experimental scheme to test the effects of PBS sham vs. Mock conditioned media on brain and lung tissues. (**B–C**) IHC for TNFα, IL10, and CD73 markers and H&E of mouse brain in B and lung in C under the various treatment conditions (PBS and Mock CM). (**D**) IHC for Ki67 staining in mouse brain and lung tissues for the various treatment conditions as in Figure 1. IHC was quantified using ImageJ for mean staining intensity. *, **, and *** represent *p*-values of <0.05, 0.01, and 0.001, respectively, as determined by student's *T*-test. *N* = 10 mice per treatment group. 100 µm and 50 µm scale bars represent full images and image inlays, respectively.

A

	Mock	8119	230	C3H	8119-C3H	230-C3H
TNFα	0.826	0.25	5.58	3.1	5.49	6.48
CD68	0.9	0.88	1.02	1.04	1.24	1.31
IL23α	1.12	6	0.85	5.63	8.11	5.28
NOS2	1.3	0.742	0.887	1.25	1.96	2.23
CCL4	0.722	0.207	0.91	0.878	6.14	5.76
IL10	1.25	0.285	10.41	2.3	159	105.4
CD163	nd	nd	nd	nd	nd	nd
MRC1	1.13	0.525	1.69	2.2	0.52	2.04
TGFβ	0.891	0.689	0.793	1.12	2.46	2.27
CCL1	1.12	2.87	1.16	1.07	1.52	1.85

B

	Mock	67NR	4T1	C3H	67NR-C3H	4T1-C3H
TNFα	0.826	1.6	0.578	3.1	3.14	2.22
CD68	0.9	0.864	0.882	1.04	0.867	0.825
IL23α	1.12	2.51	0.502	5.63	5.35	0.952
NOS2	1.3	1.12	0.638	1.25	1.28	1.26
CCL4	0.722	3.33	0.913	0.878	6.24	4
IL10	1.25	2.08	0.58	2.3	5.64	1.29
CD163	nd	nd	nd	nd	nd	nd
MRC1	1.13	3.16	1.24	2.2	2.1	2.96
TGFβ	0.891	1.39	6.12	1.12	5.35	0.986
CCL1	1.12	1.01	1.42	1.07	0.65	1.62

C

	Mock	67NR	4T1	C3H	67NR-C3H	4T1-C3H
TNFα	0.755	1.46	10.1	10.6	3.7	4.9
CD68	2.05	3.13	2.98	3.84	2.91	3.57
IL23α	0.667	5.17	2.82	1.75	2.81	2.61
NOS2	1.51	2.37	1.22	nd	1.72	2.61
CCL4	3.74	3.53	2.89	28.1	8.56	9.51
IL10	0.945	2.52	3.57	9.33	12.3	12.6
CD163	2.9	10.2	13.6	62.9	137	167
MRC1	3.85	1.28	1.86	2.2	0.292	1.4
TGFβ	0.875	2.55	2.1	1.8	2	2.41
CCL1	1.34	1.14	5.63	0.16	1.48	1.56

D

	Mock	8119	230	C3H	8119-C3H	230-C3H
TNFα	0.754	9.26	19.6	10.58	9.42	9.91
CD68	2.05	4.22	6.93	3.84	7.39	7.55
IL23α	0.667	6.55	8.07	1.25	10.22	4.12
NOS2	1.51	8.39	9.04	nd	1.4	nd
CCL4	3.75	63.9	118	28.1	20.5	47.1
IL10	0.945	14.6	28.4	9.33	13.54	13.7
CD163	2.9	70.4	356	62.9	129	132
MRC1	3.85	6.92	2.87	2.2	5.77	2.6
TGFβ	0.875	19.7	13	1.8	18.2	17.2
CCL1	1.34	0.948	0.882	0.16	1.67	1.71

Supplementary Figure 2: qPCR RQ data for gene expression analysis in association with Figure 3. (**A–D**) The same heat maps as shown in Figure 3B–3E, but with overlayed RQ values from the qPCR.

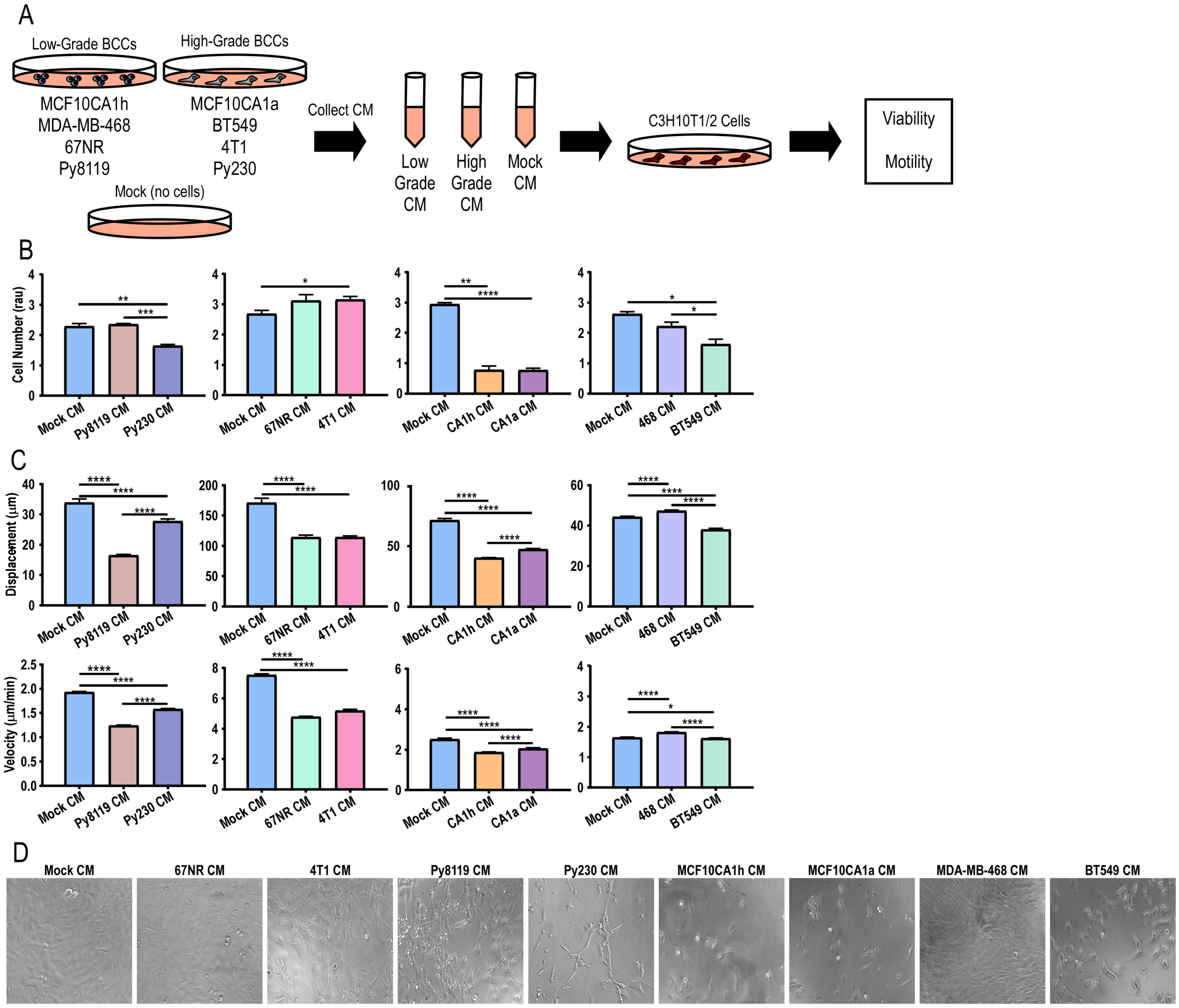

Supplementary Figure 3: Mouse and human breast cancer cell conditioned media effects on MSC viability and migration. (**A**) Experimental scheme to test the effect of both mouse and human breast cancer cell conditioned media on C3H10T1/2 MCSs. (**B**) CellTiter 96® AQueous One Solution (Promega) was used to determine C3H10T1/2 viability. Cells were cultured in mouse (Py230/Py8119, 4T1/67NR) or human (CA1h/CA1a, MDA-MB-468/BT549) conditioned medias for 96 hours on collagen or fibronectin, respectively. Absorbance was measured at 490 nm and the relative absorbance units (rau) represent relative cell viability or cell number. (**C**) C3H10T1/2 cells plated under the same conditions and treated with the same groups of breast cancer cell conditioned medias. Phase-contrast brightfield images were captured over 24 hours at 10-minute intervals. Images were quantified for displacement and velocity as described in the materials and methods. (**D**) Representative images of C3H10T1/2 cells at the 96-hour viability time point in relation to B. Mock CM was collected with each breast cancer cell media group and is not the same across all comparisons.

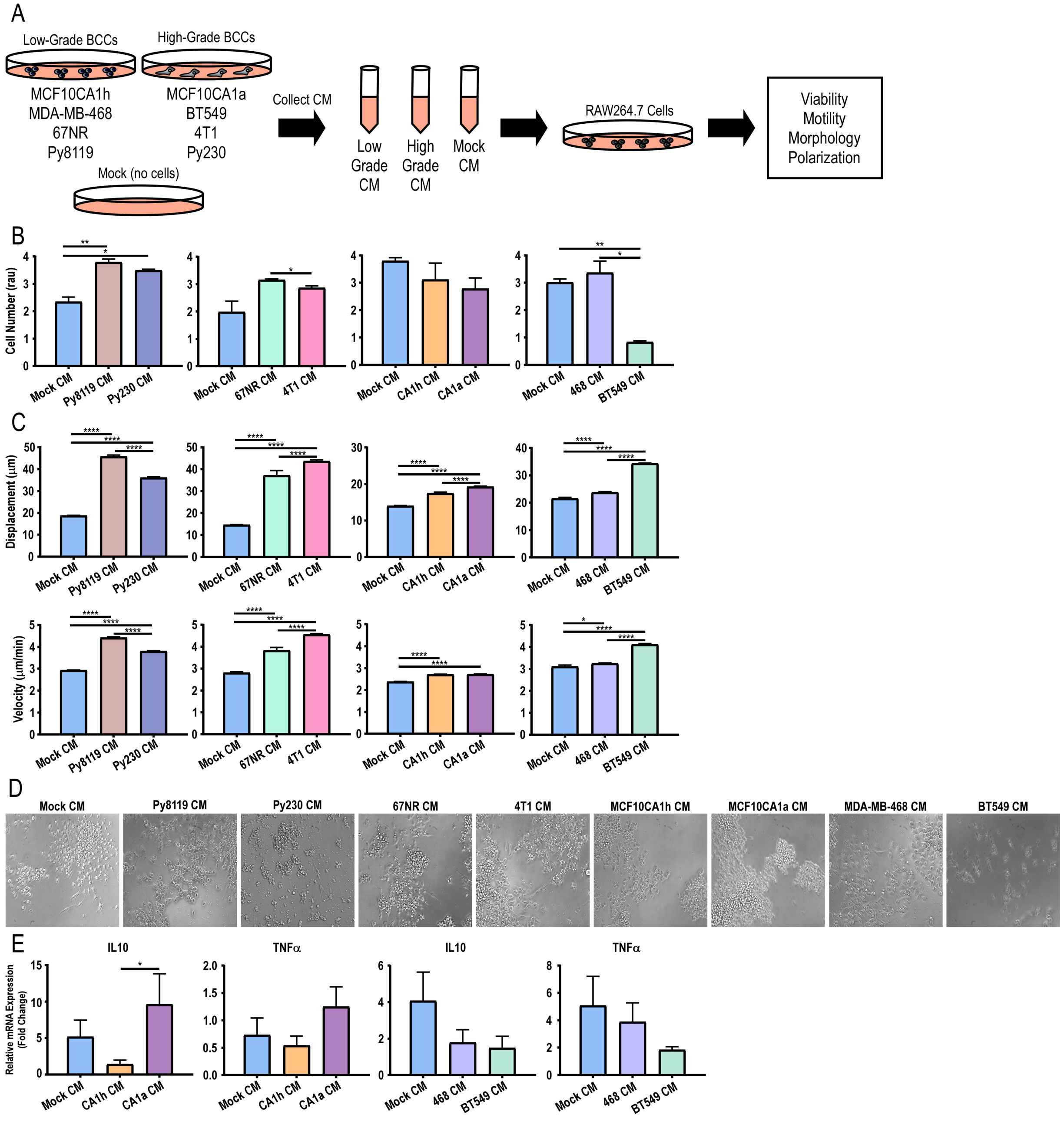

Supplementary Figure 4: Mouse and human breast cancer cell conditioned media effects on macrophage viability, migration and polarization marker expression. (**A**) Experimental scheme to test the effect of both mouse and human breast cancer cell conditioned media on RAW264.7 macrophages. (**B**) CellTiter 96® AQueous One Solution (Promega) cell viability/proliferation assay was used to determine RAW264.7 viability or relative cell number. Cells were cultured in both mouse (Py230/Py8119, 4T1/67NR) or human (CA1h/CA1a, MDA-MB-468/BT549) breast cancer cell conditioned for 96 hours on either collagen or fibronectin, respectively. Absorbance was measured at 490 nm and the relative absorbance units (rau) represent cell viability. (**C**) RAW264.7 cells plated under the same conditions and treated with the same groups of breast cancer cell conditioned medias. Phase-contrast brightfield images were captured over 24 hours at 10-minute intervals. Images were quantified for displacement and velocity as described in the materials and methods. (**D**) Representative images of RAW264.7 cells at the 96-hour viability time point in relation to B. (**E**) qPCR RQ values for IL10 and TNFα expression in RAW264.7 cells cultured in human breast cancer cell conditioned media. Mock CM was collected with each breast cancer cell media group and is not the same across all comparisons.

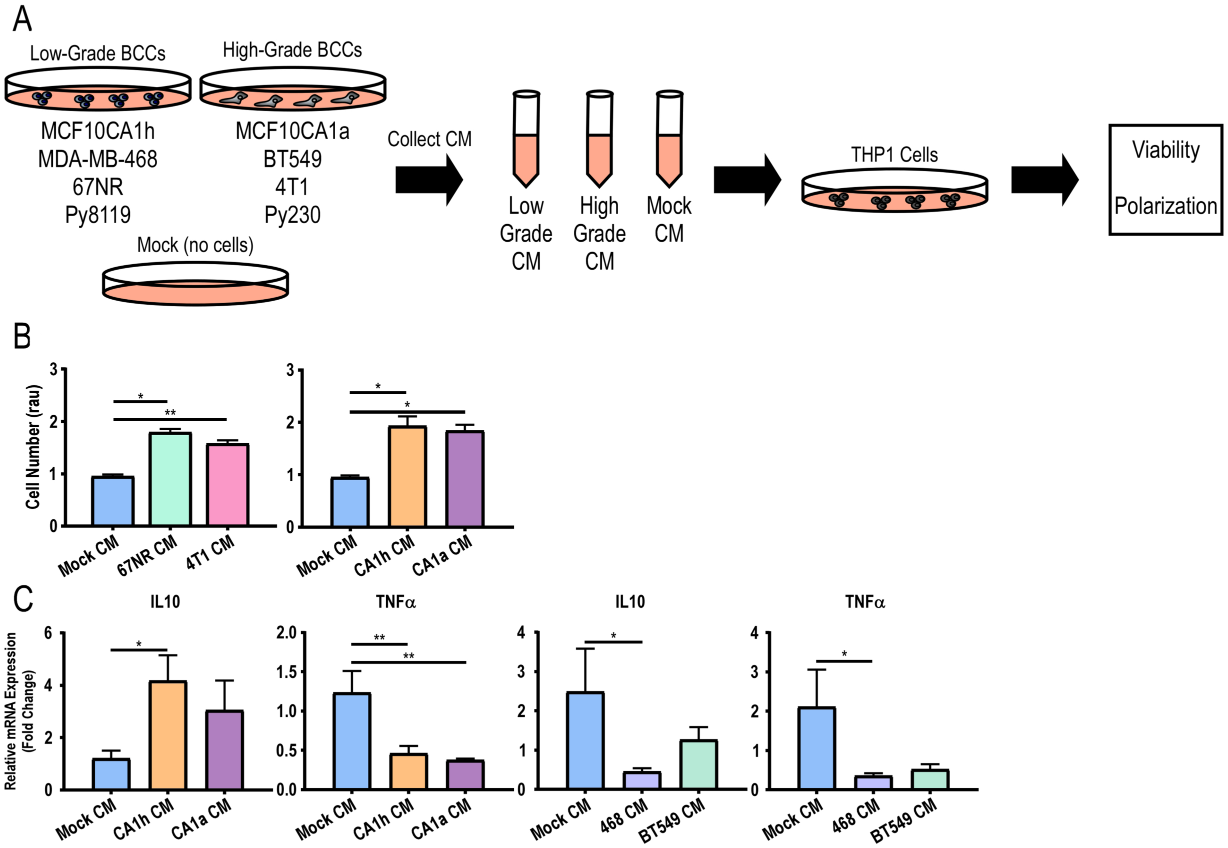

Supplementary Figure 5: Mouse and human breast cancer cell conditioned media effects on monocyte viability and polarization marker expression. (**A**) Experimental scheme to test the effect of both mouse and human breast cancer cell conditioned media on THP1 monocytes. (**B**) CellTiter 96® AQueous One Solution (Promega) cell viability/proliferation assay was used to determine THP1 viability or relative cell number. Cells were cultured in both mouse (4T1/67NR) or human (CA1h/CA1a) breast cancer cell conditioned for 96 hours on either collagen or fibronectin, respectively. Absorbance was measured at 490 nm and the relative absorbance units (rau) represent cell viability. (**C**) qPCR RQ values for IL10 and TNFα expression in THP1 cells cultured in human breast cancer cell conditioned media. Mock CM was collected with each breast cancer cell media group and is not the same across all comparisons.
